# A prospective study of decline in lung function in relation to welding emissions

**DOI:** 10.1186/1745-6673-3-6

**Published:** 2008-02-26

**Authors:** Sigve W Christensen, Jens Peter Bonde, Øyvind Omland

**Affiliations:** 1 Department of Occupational Medicine, Aalborg Hospital, Århus University Hospital, Aalborg, Denmark; 2 Department of Occupational Medicine, Århus Hospital, Århus University Hospital, Århus, Denmark; 3 Institute of Public Health, Dept. of Occupational and Environmental Medicine, University of Århus, Århus, Denmark

## Abstract

**Background:**

Numerous cross-sectional studies have reported reduced lung function among welders but limitations of exposure assessment and design preclude causal inference. The aim of this study was to investigate if long-term exposure to welding fume particulates accelerates the age-related decline in lung function.

**Methods:**

Lung function was measured by spirometry in 1987 and 2004 among 68 steel welders and 32 non-welding production workers. The decline in forced expiratory volume (FEV_1_) was analysed in relation to cumulated exposure to fume particulates among welders during the follow-up period.

**Results:**

Among smokers the decline in FEV_1 _through follow-up period was in average 150 ml larger among welders than non-welders while the difference was negligible among non-smokers. The results did not reach statistical significance and within welders the decline in lung function was not related to the cumulated welding particulate exposure during follow-up period

**Conclusion:**

Long-term exposure to welding emissions may accelerate the age-related decline of lung function but at exposure levels in the range of 1.5 to 6.5 mg/m^3 ^the average annual excess loss of FEV_1 _is unlikely to exceed 25 ml in smokers and 10 ml in non-smokers.

## Introduction

Welding of metals may cause substantial exposure to fume particulates and gases [1,2]. The main components of welding emissions are oxides of metals due to contact between the oxygen in the air and the vaporised metals. The fume particulates and gases including ozone and nitrogen oxides may cause inflammation and oxidative damage to the airways [3,4]. Such processes are thought to be closely linked to the pathogenesis of chronic obstructive pulmonary disease [COPD [5,6]]. It is well documented in clinical series that specific types of welding operations as welding stainless steel may cause occupational asthma [7] and welding in general seems related to a moderately increased risk of lung cancer [8]. However, according to a recent review "incomplete information still exists regarding the causality and possible underlying mechanisms associated with welding fume inhalation and pulmonary disease [9]."

Analyses of short term exposure to welding fumes and gases have indicated an influence on lung function in cross-shift studies [10-12] while others have not observed such effects [13]. Data from population based studies [14-16] and cross-sectional studies of working populations [17-20] have indicated a long-term effect on lung function from exposure to welding fumes and gases although the evidence is not entirely consistent [21]. Others have suggested an interaction between smoking and welding exposure on the prevalence of pulmonary impairment [22].

Only few longitudinal studies in welding populations have been published, and results have not been unambiguous [10,23,23,24]. In 2004 we re-examined the lung function in a cohort of welders and blue-collar workers examined in 1987 in a fertility study [25]. The longitudinal analyses presented here take advantage of a comprehensive assessment of exposure to welding fume particulates. The objective was to examine if exposure to welding fume particulates is a risk factor for an accelerated decline in lung function.

## Methods

### Study population

In 1987 the lung function was measured in 94 stainless steel and mild steel welders and 49 manual workers that were not exposed to welding emissions [25]. The latter group consisting of turners, mechanics, electricians, and unskilled labourers were all engaged at the same six working sites as the welders. Among the 143 participants from the 1987 survey 4 were deceased and two were immigrated in 2004. The remaining 137 men were invited in writing to participate in a follow-up study. Six declined participation, thirty-one did not respond while 100 (69.9%) participated in the second survey [68 stainless steel and mild steel welders (72.3%) and 32 non welders (65.3%, Table 1)]. Demographic characteristics and smoking habits in 1987 were slightly different among the 100 men that participated in the 2004 follow-up compared to the 43 men that did not participate (Table 1). Those not participating were younger, smoked less and had a lower prevalence of respiratory disease. The Ethics Committee of Health Science in Aalborg County, Denmark, approved the study and all participants gave their written consent.

**Table 1:** Characteristics of the study population.

	Participants in follow-up		Non-participants
	Welders N = 68	Non-welders^1 ^N = 32	N = 43

Age at enrollment in 1987, years, mean (range)	33.2 (19–55)	34.9 (21–58)	30.8 (18–53)
Height, cm, mean (range)	178 (160–190)	179 (171–193)	
Weight, kg, mean (range)	87.2 (65–124)	84.4 (70–125)	.
Tobacco smoking, n (%)			
Never-smoker	27 (40)	10 (31)	14 (33)
Ex-smoker in 1987	9 (13)	4 (13)	6 (14)
Current smoker in 1987	32 (47)	18 (56)	23 (53)
Current smoker in 2004	19 (28)	11 (34)	.
Packyears^2^, mean (range)			
Until 1987	13 (2–35)	12 (2–34)	8.0 (2.5–27)
1987–2004	11 (1–25)	8 (1–30)	.
COPD, n (%)^3^	9 (13)	2 (6)	1 (2)
History of atopy, n (%)	9 (13)	5 (16)	.
Alfa-1-antitrypsin (serum), mg/dl, mean (range)	142 (90–220)	138 (70–210)	.
Forced expiratory volume 1 sec (FEV_1_)^4^			
Liter/second, mean (range)	4.39 (3.0–5.8)	4.41 (3.3–6.0)	4.53 (3.0–6.0)
% of population average (range)	106 (82–126)	106 (82–130)	108.1 (88–138)
Forced expiratory capacity (FVC)^4^			
liter, mean (range)	5.39 (3.6–7.1)	5.54 (4.2–7.3)	5.55 (3.7–6.9)
% of population average (range)	107 (81–130)	108 (89–133)	109 (86–138)

1) A non-welder is defined as a blue-collar worker that did not perform welding operations during the follow-up period from 1987 through 2004.

2) Values given for smokers only. A pack includes 20 cigarettes or approximately 18 gram of tobacco.

3)Chronic obstructive lung disorder, FEV1/FVC < 0.70.

4) Spirometric measurements in 1987.

### Questionnaire

We obtained comprehensive information on demographic variables, characteristics of welding work and smoking habits for the period 1987 until 2004 by face-to face interviews in 1987 and 2004 (Table 1). The same questionnaire was used in 1987 and 2004, and the questions on respiratory diseases and symptoms were based upon the modified British Medical Research Council questionnaire [26]. Additional questions on history of atopy were also asked. Atopy was defined by positive answer to at least one of the three questions [27]: Have you ever have had asthma? Have you ever have had any kind of allergy to the nose, such as hay fever? Have you ever have had eczema or some kind of skin allergy?

### Welding exposure

A consecutive list of employments for all metalworkers from primary school through 2004 was collected by interview. For each employment we extracted information on the first and last calendar year, the type of metal in which the welding was performed (mild steel, stainless steel, chromium alloyed steel and aluminum), welding method [manual metal arc (MMA), metal active gas (MAG) and tungsten inert gas (TIG)], welding intermittence (average number of hours during a work shift), use of local exhaust ventilation (yes/no/sometimes), and welding in confined spaces (yes/no/sometimes). We reorganized the data to obtain welding characteristics by decades (1950–59, 1960–69, 1970–79, 1980–89, 1990–99 and 2000–2004) in order to estimate lifelong cumulated exposure to welding fume particulates based upon a large Danish welding exposure database that provides ambient air particulate concentration by decade, type of steel, welding method, hours welding a day, exhaust ventilation and welding in confined spaces. The exposure matrix include 364 cells each providing the geometric mean exposure to welding fume particulates for a specific welding process.

The welding exposure matrix is based upon 1.106 measurements (786 MMA mild steel, 58 MMA stainless steel, 51 TIG stainless steel, and 152 MAG mild steel) of particulates in ambient air samples collected in filters placed in the breathing zone behind the welding helmet [28]. The measurements were performed in the Danish welding industry from 1971 – 1985 by the Danish Welding Institute and the Danish Occupational Health Institute and are considered to provide reliable and representative exposure data. The database provides the geometric mean value of the welding process specific particulate exposure (mg/m^3^). The process specific exposures in decades before 1970 were estimated by extrapolation based upon the declining trend of exposures that were observed from 1971 to 1985 for all types of welding processes. Very few welding exposure measurements have been carried out in Denmark since 1989. For purposes of the present exposure assessment we assumed that the average exposure levels have not changed from 1989 and onwards. However, in an alternative approach we also estimated exposures in 1990–1999 and 2000–2004 based upon linear extrapolation of exposures from 1971–85 and thus arriving at lower exposure estimates.

Using the welding exposure matrix and the corresponding interview data on decade, type of steel, welding method, intermittence, exhaust ventilation, and welding in confined spaces we computed the product of number of years and particulate concentration in each decade and summed over all decades to arrive at a summary measure of total lifelong welding exposure (mg/m3*years), Table 2. No exposure data were available for welding of alloyed steel at one plant. Estimates were based upon the corresponding values for mild steel. Likewise, we used exposure data for TIG stainless steel welding to estimate exposure conferred by TIG welding on aluminum in six welders. The exposure matrix does not account for bystander and background exposure levels. This will result in a disproportionately underestimation of exposure among workers with low welding intermittence relative to workers welding most of the work shift. Therefore, the cumulative exposure estimates were refined by addition of a term indicating background exposure. According to full shift measurements performed in 1987 at 3 of the work places, the geometric mean bystander exposure among mild steel welders was 1.2 mg/m^3 ^(SD) and among stainless steel welders 0.8 mg/m^3 ^(SD). For each year in a welding shop we added bystander exposures corresponding to the numbers of hours during a work shift that welding was not performed (and therefore not included in the process specific exposure estimate). We did not accounted for the few metal workers using infrequent welding methods as welding robots and tube trod.

**Table 2:** Distribution of estimated cumulative exposure to welding fume particulates by type of steel and main welding technology among ever-welding Danish metal workers.

		N Welders	Years welding, mean (STD)	Geometric mean particulate exposure, mg/m^3^	Geometric mean (min-max), mg/m^3 ^* years, 1950–2004^1^	Geometric mean (min-max), mg/m^3 ^* years, 1987–2004^1^
Mild steel	manual metal arc (MMA)	37	7.5 (6.1)	4.1	31 (8–103)	17 (7–61)
	metal active gas (MAG)	39	12.4 (9.7)	3.2	40 (7–134)	26 (9–50)
Stainless steel	manual metal arc (MMA)	1	5.0	3.0	15 (15–15)	0
	tungsten inert gas (TIG)	28	15.1 (10.9)	1.4	22 (3–63)	14 (3–26)
Alloyed steel	manual metal arc (MMA)	20	16 (12.4)	6.5	105 (8–478)	42 (7–166)
	tungsten inert gas (TIG)	13	11.6 (7.6)	1,5	17 (2–34)	12 (2–23)
Aluminum	tungsten inert gas (TIG)	7	4.8 (5.5)	2.3	11 (3–22)	11 (3–22)
All particulates		76	.	.	88 (4–499)	37 (3–166)^2^

1) estimates based upon the assumption that exposures levels have not declined from 1989 onwards.

2) includes 68 men performing welding tasks during at least one appointment from 1987 through 2004.

### Validation of exposure estimate

As part of the baseline survey in1987 the Danish Welding Institute performed 44 full shift measurements of particulates in the breathing air in randomly selected welders within the frequent welding processes (MMA MS, MAG MS, and TIG SS) in three plants from which welders for this survey were recruited. This hygienic survey also included 12 full-shift stationary measurements of particulates in the vicinity of the welding processes to estimate bystander exposure. We validated the exposure estimate by comparing the welding process specific results of the 1987 measurements with the exposure levels predicted by the exposure assessment model taking decade and welding characteristics into account. There was a high agreement in welding fume measurements in 1987 and the predicted exposure levels based upon the welding fume exposure matrix and the interview information obtained in 2004. The geometric means for mild steel welding with MMA technology were identical (3.5 mg/m^3^) while the estimated values for mild steel welding with MAG technology and stainless steel welding with TIG technology were slightly higher than the measured values (Table 3).

**Table 3:** Full shift average exposure to welding fume particulates measured in air samples collected among 44 randomly selected welders in 1987 in comparison with estimated exposures based upon the welding exposure matrix.

	Measurements in 1987		Estimated exposures	
	n	Geometric mean mg/m^3^	n	Geometric mean mg/m^3^

Mild steel manual metal arc (MMA)	14	3.5	20	3.5
Mild steel metal active gas (MAG)	15	3.0	12	3.2
Stainless steel, inert tungsten gas (TIG)	15	1.1	15	1.3

### Lung function

The same dry wedge spirometer (Vitalograph, Buckingham, UK) was used in 1987 and 2004. On days testing lung function the spirometer was calibrated with a 1 L Vitalograph precision syringe (AT No 20408) before and after the sessions to ensure correct measures. Forced expiratory volume in one second (FEV_1_) and forced expiratory capacity (FVC) were recorded in accordance with American Thoracic Society guidelines (24). Lung function data were analysed in absolute figures as well as percentage predicted from gender, age, and height specific Danish reference data [29]. Baseline measurements had not been undertaken in 8 of the non-welders that participated in the follow-up.

### Measurements of alfa-1-anitrypsin

Blood samples were collected in a dry tube and analysed the same day or frozen at -20°C until analysed using a Beckman Coulter Immage^® ^kit, Beckman Coulter, CA, USA. All measurements of alfa-1-antitrypsin were within normal range (88–174 mg/dl).

### Statistical analysis

We analyzed the data by two different approaches. In the first approach we used the absolute measured values of lung function to examine the change in FEV_1 _and FVC across the 18 years from 1987 to 2004 in welders and non-welders. The intra-individual change of lung function was analysed by multiple linear regression methods that adjusted for the following base-line characteristics: height (squared), age, FEV_1_, atopy (history of asthma, allergic rhinitis or demonstration of IgE antibodies against standard allergens, yes/no), and serum concentration of alpha-1-antitrypsin (continuous variable). Moreover models were adjusted by weight at follow-up (not measured at baseline). Variables were kept in the models if deletion of the variable changed the adjusted decline in lung function by more than 10%.

Since the reference group of non-welders was of limited size we also in a second approach analyzed the change in FEV_1 _expressed as a percentage of the average gender, age, and height specific values that were obtained from a Danish reference material [29]. Again the change in percentage of predicted FEV_1 _from 1987 through 2004 was examined in welders as well as non-welders.

In both approaches using absolute and relative lung function values, respectively, we subsequently performed analyses of the decline in lung function according to our estimate of the individual cumulated welding particulate exposure during entire life and during the follow up period 1987 to 2004. The latter were entered into the models as (i) a continuous variable, (ii) participant quartiles, or (iii) four categories with equidistant exposure contrasts. These analyses only included welders in order to examine exposure-response relations within welders separately from effects attributable to differences between welders and non-welding metal workers, turners and electricians. Moreover, these analyses used the cumulative particulate exposure during the entire working life as well as welding exposure contributed during the period from 1987 to 2004 only. Finally, we repeated all analyses using estimates of exposure contributed by MS MMA, MS MAG, and SS TIG only to distinguish possible effects of specific contaminants related to the welding process – for instance ozone related to gas shielded welding techniques. All analyses were performed by SAS 9.1 software [30].

## Results

The decline in FEV_1 _among the 100 manual workers averaged 688 ml (SD 390 ml) across the 18 years from the men were in average 33 years until they became in average 50 years old. This is corresponding to an annual decline of 38 ml.

FEV_1 _values declined on average 856 ml in welders that smoked tobacco at the start of the 18-year follow-up and 704 ml among smoking non-welders (Table 4). This larger decline among the welders amounting some 150 ml did not reach statistical significance and differences among welding and not welding non-smokers were even smaller (Table 4). Very similar findings were observed when considering changes in relative FEV_1 _values (Table 5). In smokers, welders had a larger decline than non-welders, but the difference was not significant and differences among non-smokers were rather marginal.

**Table 4:** Decline in lung function from 1987 through 2004 among 68 welders and 24 non-welders stratified on smoking status at baseline. Crude and adjusted average difference (2004 minus 1987 values) and 95% confidence limits (CL).

	**Measure of lung function**	**n**	**Baseline values**	**Average crude decline**	**Average adjusted^**1 **^decline**	**Difference in decline [welders – referents, (95% CL)]**
**Smokers**						
**Welders**	FEV_1_, L	32	4.26 L	0.85 L	0.856 L	0.15 L (-0.15 to 0.45)
**Non welders**	FEV_1_, L	12^2^	4.39 L	0.72 L	0.704 L	Reference
**Non-smokers**						
**Welders**	FEV_1_, L	36	4.50 L	0.56 L	0.559 L	-0.05 L (-0.28 to 0.18)
**Non welders**	FEV_1_, L	12^2^	4.43 L	0.61 L	0.607 L	Reference

^1 ^Adjustment for baseline characteristics (age, height^2^, FEV1, alfa-1-antitrypsin), body weight measured at follow-up and cigarette pack years during the follow-up period (smokers only).

^2 ^Eight non-welders with missing spirometric measurements

**Table 5:** Change in predicted FEV_1_-values from 1987 through 2004 among 68 welders and 24 non-welders.

	**N**	**Predicted FEV_**1 **_in 1987**	**Predicted FEV_**1 **_in 2005**	**Change in predicted values, mean (95% CL)**	**Difference in decline [welders – referents, (95% CL)]**
**Smokers**					
**Welders**	32	104 %	96 %	-8.1 (-12.6 to -3.6)	3.8 (-4.4 to +12.4)
**Non welders**	12^1^	107 %	103 %	-4.3 (-11.7 to +3.0)	Reference
**Non-smokers**					
**Welders**	36	109 %	109 %	-0.1 (-3.4 to +3.3)	1.6 (-5.1 to +8.4)
**Non welders**	12^1^	105 %	107 %	-1.6 (4.3 to +7.4)	Reference
**All**					
**Welders**	68	107 %	103 %	-3.0^2 ^% (-5.5 to -0.5)	1.5 (-3.6 to +6.5)
**Non-welders**	24^1^	106 %	105 %	-1.5^2 ^% (-6.0 to +2.8)	Reference

^1 ^Eight non-welders with missing measurements of lung function at baseline

^2 ^The average change in predicted values were adjusted for FEV1 level at baseline, body weight measured at follow-up and cigarette pack-years during the follow-up period.

Among the 68 welders that did welding work during the follow-up period the decline in FEV_1 _was not related to cumulative welding particulate exposure during the follow-up period (Table 6). Analyses of FVC revealed similar findings. Separate analyses of particulate exposure conferred by welding of mild steel, stainless steel and welding with the MMA, MAG, and TIG methods, respectively, did not indicate effects on FEV_1 _or FVC (data not shown). In the latter analyses we examined exposure-response relations between the acumulated exposures during follow-up according to the specific welding technology. We also examined decline in lung function according to exposure estimates assuming a continuous fall of exposure levels from 1989 onwards but found no indications of effects of welding fume particulates. Finally, all analyses were repeated using FEV_1_/FVC ratio as the outcome. No association with welding fume exposure was demonstrated.

**Table 6:** Decline in lung function from 1987 through 2004 according to an estimate of cumulated exposure to welding fume particulates (mg/m^3 ^* years) among 68 workers with welding tasks from 1987 through 2004.

	**Measure of lung function**	**n**	**Baseline values**	**Average crude decline**	**Average adjusted^**1 **^decline**	**Difference in decline [high-level exposed – low-level exposed, (95% CL)]**
**Smokers**						
0–25 mg/m^3^*y	FEV_1_, L	8	4.24 L	0.97 L	1.03 L	Reference
26–50 mg/m^3^*y	FEV_1_, L	12	4.16 L	0.77 L	0.73 L	-0.29 L (-0.84; 0.25)
51+ mg/m^3^*y	FEV_1_, L	12	4.37 L	0.85 L	0.85 L	-0.17 L (-0.69; 0.34)
**Non-smokers**						
0–25 mg/m^3^*y	FEV_1_, L	15	4.53 L	0.55 L	0.56 L	Reference
26–50 mg/m^3^*y	FEV_1_, L	13	4.55 L	0.54 L	0.54 L	-0.01 L (-0.32; 0.30)
51+ mg/m^3^*y	FEV_1_, L	8	4.36 L	0.60 L	0.59 L	0.03 L (-0.33; 0.40)

^1 ^Adjustment for baseline characteristics (age, height^2^, history of atopic disorder, FEV1), body weight measured at follow-up and cigarette packyears during the follow-up period.

Smokers in 1987 had a more pronounced decline in FEV_1 _through the follow-up than non-smokers: the adjusted decline in smokers was in average 280 ml (95 CL 123–437 ml, p = 0.0006)] greater than the decline among non-smokers. Moreover, the decline was significantly related to cigarette pack years through the follow-up period 1987–2004. Thus FEV_1 _declined 16 ml per pack year (SE 6.9 ml, p = 0.03). Age, height and FEV_1 _at baseline and weight at follow-up was not related to lung function decline during follow-up, but the baseline FEV_1 _was significantly related to age (lower at higher age), height (higher at higher height) and smoking (reduced among smokers), but not to welding at baseline or to cumulated exposure up to baseline (cross-sectional analysis, data not shown).

Welders were not at increased risk of accelerated decline of lung function during follow-up in comparison with non-welding metal workers (Table 5).

## Discussion

Measurements of lung function 18 years after baseline indicated a limited accelerated loss of lung function among in particular smoking welders that were compared to a reference group of non-welding metal workers, turners, and electricians enrolled from the same plants. However, differences did not reach statistical significance and within the group of welders the decline in lung function was not related to the total welding particulate exposure during the follow-up period. As expected smoking had a strong effect on FEV_1 _that in average declined some 280 ml more in smokers than in non-smokers after adjustment for the effect of welding.

Only few studies have been published that analyses effects of welding exposure on lungs in non-smokers. Meo et al found in a *cross-sectional *study of 50 non-smoking manual metal arc welders and 50 non-smoking controls a significant smaller lung function among welders exposed for more than 9 year (640 ml in FEV_1_), suggesting an independent effect of welding fume on the lungs [19].

Few *longitudinal *studies in welders addressing decline in lung function have been published (18–20). In a two-year follow-up (1996–98) from New Zealand lung function was measured in 54 TIG and MIG welders and 38 non-welders aged 40–41 years [24]. No exposure measurements were made. Smoking welders had a significantly greater annual decline in FEV_1 _(88.8 ml) than smoking referents (34.2 ml). Moreover, welders without respiratory protection or local exhaust ventilation had a greater annual decline in FEV_1 _(168.8 ml) than welders with protection (87.3 ml). The annual loss is surprisingly high, and higher than observed in the present study. Differences in severity of exposure to welding fumes, duration of exposure, welding in confined spaces, and use of protective equipment together with smoking habits may explain the apparent inconsistency. Our data are similar to the findings of Beckett et al [10]. They found an annual decline in FEV_1 _among welders of 40 ml and no overall excess decline in lung function in welders compared to control subjects during the three year of follow up. The participants were 51 MMA shipyard welders aged 32 (92% males) fabricating steel alloy structures and 54 control subjects aged 39 (draftsmen and technical aids employed at the shipyard) (83% males). No exposure measurements were reported, and it was not indicated when the study was undertaken. Chinn et al studied English shipyards workers from 1980–84 to1987–1991. In all 346 subjects participated in the follow-up (welders, caulker/burners and possibly also electricians) with an average follow-up time of 6.7 years. Age at baseline was 17–30 years. The overall annual decline in FEV_1 _was 11.1 ml. Continuing smoking was associated with increased loss in FEV_1 _and atopy in smokers further increased the loss in FEV_1_. Continued shipyard work as welders or caulker/burners was associated with enhanced deterioration in FEV_1_. The annual decline in lung function is in the same range as data from the present study. The cohort was studied a decade before ours, and this might indicate a higher exposure to welding fumes than in the present study. Moreover, shipyards welders may have worked more in confined and poorly ventilated spaces with higher risk from impairment of lung function than welders working in more well ventilated areas [28,31].

Less than 70% participated in the second survey. We cannot entirely exclude that high-level exposed workers with impaired lung function declined participation to a higher degree than others and thus introduce bias towards the null. However, the fact that the loss to follow-up was balanced in welders and non-welders and that the non-participants were younger with less tobacco smoking and respiratory symptoms do not support this hypothesis. It seems unlikely that weak associations between welding fume exposure and lung function is due to selective processes.

Our findings have limitations due to small sample size, especially for analyses of subgroups, and the small sample size reduces the ability to generalize the results. On the other hand, the present data are aggregated from longitudinal analyses with a long follow-up time and accounting for the dropouts. Although a larger study group is definitely warranted the number of person-years at-risk is far higher in this study (some 1800 person-years) than in two earlier longitudinal studies [(200–300 person-years, [10,24]] and in range with the third longitudinal study that has been undertaken so far [2350 person-years, [23]]. Finally, the number of person-years investigated in this study is in range with numbers in large randomized studies as the ISOLDE project where 759 subjects were followed in three years [2.250 person-years, [32]]. The confidence intervals indicate that effects of welding at the encountered exposure levels in this study, if any at all, are unlikely to exceed an excess annual decline of 25 ml in smokers and 10 ml in non-smokers.

Our exposure assessment is associated with some degree of misclassification and a less likely chance to detect effects of welding fume exposure on lung function, if any. Information on individual welding tasks during the entire working life was obtained up to 40 years after the work was actually performed and the accuracy of recall of details with respect to type of welding, time spent with welding, and working conditions are questionable. For instance, use of exhaust and working in confined spaces that have major impacts of welding exposure levels [28]. Moreover, use of different welding techniques and materials across short time periods can only partly be accounted for in data collection and computations. There is a large residual variation in exposure levels that the geometric mean values does not account for, even if our exposure estimate includes several major welding characteristics such as calendar time, welding technology and working conditions. This may, however, be less of a problem since this large variation is observed within welders across the work shift and across different work shifts so that group averages may provide a more reliable estimate of exposure than for instance individual point measurements [33,34]. Furthermore, background exposure, dust exposures from grinding and other work processes and less frequent welding techniques could not be accurately accounted for. Nevertheless, we found a rather good agreement between actual full shift welding fume measurements performed in 1987 and the exposure levels that was predicted by computations based upon the welding fume exposure matrix and the interview information obtained in 2004. It must be noticed that evaluation of the validity of the exposure assessment only applies to the years 1980–89 and only for some of the welding processes (Table 4). The exposure levels in 1950–1970 and from 1990–2004 are largely based upon extrapolation and therefore less reliable. In particular data on welding fume exposure past 15 years is very scanty. However, findings were the same in models based upon a continuous linear decline in exposure levels from 1989 onwards and in models assuming constant exposure levels during the follow-up period.

In conclusion, we found some indications of a limited accelerated loss in lung function after 18 years among young and middle-aged welders. Geometric mean particulate exposure was estimated to 1.5 to 6.5 mg/m^3^. As opposed to welding fume exposure smoking did significantly contribute to accelerated decline in lung function.

## Competing interests

The author(s) declare that they have no competing interests.

## Authors' contributions

OO and SC conceived the study and wrote the protocol. SC organized the data collection. JPB performed data analyses. JPB and OO drafted the paper. All authors read, revised and approved the final manuscript.
